# Aspirin in Primary Prevention: It Is Time to Start Stepping Away∗

**DOI:** 10.1016/j.jacadv.2023.100281

**Published:** 2023-03-22

**Authors:** Nishant P. Shah, Neha J. Pagidipati

**Affiliations:** a Duke Clinical Research Institute, Durham, North Carolina, USA; b Division of Cardiology, Duke Department of Medicine, Durham, North Carolina, USA

**Keywords:** aspirin, cardiovascular risk, prevention

**Figure:**
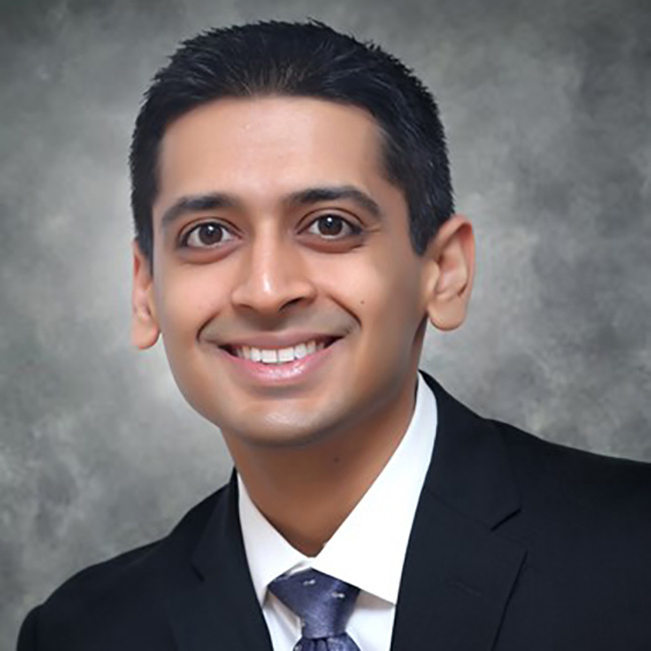

Aspirin is one of the most widely used drugs in medicine.^1^ While the benefits in secondary prevention have long been clear, the risk-benefit calculus in primary prevention is far less straightforward. Several randomized controlled trials conducted in the 1980s and 1990s showed cardiovascular benefits of aspirin in primary prevention,^1^ but more recent studies have called this benefit into question.^2^ One potential explanation for this discrepancy is that in the more recent era of statin therapy for primary prevention, the absolute risk of the population—and therefore, the absolute benefit of aspirin—has been reduced. Understanding the impact of aspirin therapy in patients on concomitant statins is highly relevant to current clinical practice and yet has been underexplored.

In this issue of *JACC: Advances*, Khan et al^3^ address this important question by assessing the impact of aspirin with and without statin therapy in patients without known atherosclerotic cardiovascular disease (ASCVD). The authors performed a meta-analysis of 16 randomized controlled trials of aspirin (at least 75 mg every other day) in adults without ASCVD. The primary outcomes were fatal and nonfatal myocardial infarction (MI) and major bleeding requiring transfusion or hospitalization or leading to death. These outcomes were assessed in patients at various ASCVD risk categories based on the Cholesterol treatment Trialist’s Collaboration framework, ranging from very low risk of an event (<5%) to very high risk (≥30%).

The authors found that among the 171,215 participants in the 16 trials, aspirin was associated with a reduction in risk of MI (risk ratio: 0.85 [95% CI: 0.77-0.95]; *P* < 0.001; I^2^ = 57%) but at the cost of a higher risk of major bleeding (risk ratio: 1.48 [95% CI: 1.32-1.66]; *P* < 0.001; I^2^ = 19%). Both the benefit and risk of aspirin were dependent on the patients’ level of ASCVD risk that the decrease in MI risk and the increase in major bleeding risk were of smaller magnitude in those at low vs high ASCVD risk. Importantly, the absolute risk reduction of MI with aspirin was lower at every level of risk when statin therapy was used concomitantly. However, the risk of major bleeding with aspirin was not impacted by statin use. Thus, in the context of statin therapy, aspirin had less absolute benefit but no reduction in risk of bleeding, making the risk-benefit calculus even less favorable for aspirin in primary prevention. With regard to the endpoints of stroke, all-cause death, and CV death, aspirin was not associated with benefit, either alone or in combination with statin therapy.

When interpreting these findings, there are some caveats to note. First, the dose of aspirin therapy varied across trials although it is reassuring that in a sensitivity analysis, there was no significant difference in risk of MI or of major bleeding with aspirin ≤100 mg/d vs >100 mg/d. Furthermore, this meta-analysis was based on aggregate data from trials with varied participants, outcomes, and follow-up. Similarly, ASCVD and bleeding risk were based on aggregate-level data, which may not accurately reflect individual-level risk.

Notwithstanding these limitations, the findings of this analysis have important implications for clinicians. Ultimately, in primary prevention patients—especially those already on statin therapy—the absolute risks of aspirin appear to outweigh the benefits at every level of risk. However, this unfavorable risk-benefit ratio is not reflected in aspirin use in the general population. Indeed, among primary prevention adults aged ≥40 years, 23.4% (approximately 29 million individuals) take aspirin for prevention of ASCVD; of these, nearly one-quarter (6.6 million individuals) do so without a clinician’s recommendation.^4^ Furthermore, nearly half of the people older than 70 years without known ASCVD report taking aspirin for primary prevention. These sobering statistics underscore the widespread prevalence of inappropriate aspirin use.

However, before we stop aspirin in all primary prevention patients, we must consider the increasing number of patients who fall in the “grey zone” between primary and secondary prevention, such as those with subclinical atherosclerosis found on imaging. Several epidemiologic studies indicate that individuals with elevated coronary artery calcification scores may have more favorable risk-benefit ratios with aspirin therapy than those without any calcification.^5^ Similarly, observational studies suggest that people with elevated lipoprotein(a) may benefit from aspirin therapy, even in the absence of known ASCVD.^6^ However, rigorous, randomized trials of aspirin therapy in these high-risk populations are lacking. Thus, shared decision-making and personalized risk classification are still of utmost importance when deciding about any therapy for ASCVD prevention.

Overall, the analysis by Khan et al^3^ provides added insights into the decreasing role of aspirin therapy in primary prevention, helping to guide our discussions with patients who are understandably confused about whether or not they should take aspirin daily. This study highlights that while decision-making about preventive therapies should be tailored to and made in conjunction with patients, we need to start stepping away from the routine use of aspirin therapy in primary prevention, especially in the modern era of statins.

## Funding support and author disclosures

Dr Shah has received research grants from 10.13039/100002429Amgen, 10.13039/100005565Janssen, and 10.13039/100000002National Institutes of Health; and is a consultant/advisor for Esperion, Amgen, and Norvartis. Dr Pagidipati has received research grants from 10.13039/100002429Amgen, 10.13039/100004325AstraZeneca, Baseline Study LLC, 10.13039/100001003Boehringer Ingelheim, 10.13039/100006513Duke Clinical Research Institute, Eggland’s Best, 10.13039/100004312Eli Lilly & Company, 10.13039/100004336Novartis, Novo Nordisk Pharmaceutical Company, Sanofi-S.A, and Verily Sciences Research Company; and is a consultant/advisor for AstraZeneca, Boehringer Ingelheim, Esperion Therapeutics, Eli Lilly & Company, and Novo Nordisk Pharmaceutical Company.
